# Meeting of the Mississippi State Medical Association

**Published:** 1873-05

**Authors:** S. V. D. Hill


### MEETING OF THE MISSISSIPPI STATE MEDICAL ASSOCIATION.

*Editors Southern Medical Record:*

Having just returned from the annual meeting of our State Medical Association, I cannot resist the inclination to tell your readers something about it, with the hope of stimulating a deeper interest in such organizations. We met at Vicksburg on the 2d of April, ultimo, and had an attendance of one hundred and ten doctors, from every portion of the State. I have never seen a more dignified, intelligent body of men on any occasion nor for any purpose, nor more harmony, good feeling and zeal than were exhibited there. A large number of papers, sufficient to fill a volume of three hundred pages, were presented and discussed; the most of them highly interesting and instructive. The address of the President, Dr. Gallaway, (one of your associates) was able, appropriate and eloquent. He gave us much wholesome advice, suggested many reforms, and urged the correction of abuses that should not be neglected. The habit of M.D.'s and D.D.'s giving certificates to the virtues of patent medicines was condemned in unmeasured terms, and his views on this subject were sustained by the Association. In fact, we made an unmerciful attack upon quacks, patent medicine vendors, and irregulars generally. We sewed them up with our *Taylor*, tore them to pieces with our *Lyon*, hammered them with our *Smith*, and sent them up salt river by our *Craft*.

The oration of Dr. Lyon on the evening of the first day, to a large, fashionable and intelligent audience of ladies and gentlemen, was a splendid effort—chaste, elegant and scholarly, abounding in thrilling incidents of professional life; details of progress and improvement in medicine; duties of the public to physicians, and *vice versa*, with striking and impressive illustrations. The address was listened to with marked attention all the way through, and lofty encomiums were heaped upon the Doctor from every source.

It would require too much space to even allude to all the papers presented. Dr. Compton, of Jackson, read a highly interesting and amusing essay upon the "Duties of Physicians as Experts." If his advice was generally heeded, there would be less sparring between lawyers and doctors, and the latter gain more and lose less than they have been in the habit of doing when called as witnesses in court.

Dr. Hunt, of Vicksburg, presented a negro man from whom he had resected ten inches of the lower extremity of the tibia for osteo-myilitis, with complete recovery; the negro was then walking well, and resting the weight of his body upon the fibula alone, without crutches.

Dr. Lyon reported a case of reproduction of the anterior half of the penis. The patient, eighteen years of age, had variola; the glans became gangrenous, and gradually half of the body; a line formed, granulations sprang up, and the plastic process continued until the organ complete (urethra and all) was reproduced. The decayed mass remained as a lateral appendage until the Doctor separated it with his scissors. This was preserved and presented to the Association, as well as photographs of the new organ; both, as regards appearance, confirming the history of the case.

Dr. Hicks, of Vicksburg, presented a new splint, as a substitute for Smith's anterior. It consisted of a piece of wooden hoop or common white-oak the length of the limb and about an inch wide. This is to be bandaged to the front aspect of the limb, adapting it to the inequalities, and otherwise managing it as with Smith's. He claims for it (and I think justly) lightness, easy management, cheapness, and being always at hand.

Dr. Cage, of Canton, reported a case of ovariotomy in which no ligature, clamp or other means of constricting the pedicle was employed. The tumor was detached with a knife, and after all bleeding—which was slight—ceased, the pedicle was returned to the abdomen, and the patient recovered in good time. Might not this plan be adopted as the rule in all cases, thereby saving the patient the danger of a foreign body in the abdomen?

Dr. Capers, of Vicksburg, made an excellent report on surgery, which I have not space to notice extensively. He strongly advocates the superiority of knee-joint amputations, and mentioned a number of interesting cases.

We were entertained at the homes of the medical men of Vicksburg. They would not permit any of us to pay a hotel bill; besides, did everything possible to make our stay pleasant and profitable. Vicksburg should be proud of her medical faculty, for they are above the average in all that constitutes excellence in doctors and men. Ah! the splendid dinners we had—the wit, the wine, the sentiment and soul.

S. V. D. Hill, M.D.

